# Supplementary material for: Enzymatic Synthesis of New Acetoacetate–Ursodeoxycholic Acid Hybrids as Potential Therapeutic Agents and Useful Synthetic Scaffolds as Well
Source: Molecules. 2024 Mar 15;29(6):1305. doi: 10.3390/molecules29061305 (PMC10975173; doi:10.3390/molecules29061305)

Enzymatic synthesis of new acetoacetate-ursodeoxycholic acid hybrids as potential therapeutic agents and useful synthetic scaffolds as well.

Valentina Venturi [1,†], Elena Marchesi [2,†], Daniela Perrone [1], Valentina Costa [1], Martina Catani, Simona Aprile [2], Lindomar Alberto Lerin [2], Federico Zappaterra [2], Pier Paolo Giovannini [2,*] and Lorenzo Preti [1]

[1]Department of Environmental and Prevention Sciences, University of Ferrara, Ferrara, 44121, Italy.

[2]Department of Chemical and Pharmaceutical Sciences, University of Ferrara, 44121 Ferrara, Italy.

Supplementary Materials

Contents

Biocatalyst reuse	S1
Determination of the conversion by ^{1}H NMR analysis	S2
^{1}H- and ^{13}C-NMR spectra of compound **3a**	S3
^{1}H- and ^{13}C-NMR spectra of compound **3b**	S4
^{1}H- and ^{13}C-NMR spectra of compound **3c**	S5
^{1}H- and ^{13}C-NMR spectra of compound **4a** (diastereomeric mixture)	S6
^{1}H-NMR spectra of single diastereoisomers of compound **4a**	S7
UPLC-MS chromatogram and HRMS of compound **3a**	S8
UPLC-MS chromatogram and HRMS of compound **3b**	S9
UPLC-MS chromatogram and HRMS of compound **3c**	S10
UPLC-MS chromatogram and HRMS of compound **3d**	S11
UPLC-MS chromatogram and HRMS of compound **4a**	S12

Figure S1: Biocatalyst reuse

The ursodeoxycholic acid **1**, (250 mg, 0.634 mmol) and methyl acetoacetate **2** (370 mg, 3.19 mmol) were dissolved in *t*-butanol (5.0 mL). Lipuraflex (50 mg, 500 U) was added, and the mixture was shaken at 50 °C for 24 h. The biocatalyst was removed by filtration and added to a fresh reaction mixture. The filtrate was evaporated under reduced pressure to remove the solvent and the excess of **2** and the residue was submitted to ^{1}H NMR analysis to determine the conversion of **1** into its 3α-acetoacetoxy derivative **3a**.

Activity of Lipura Flex in consecutive reaction cycles

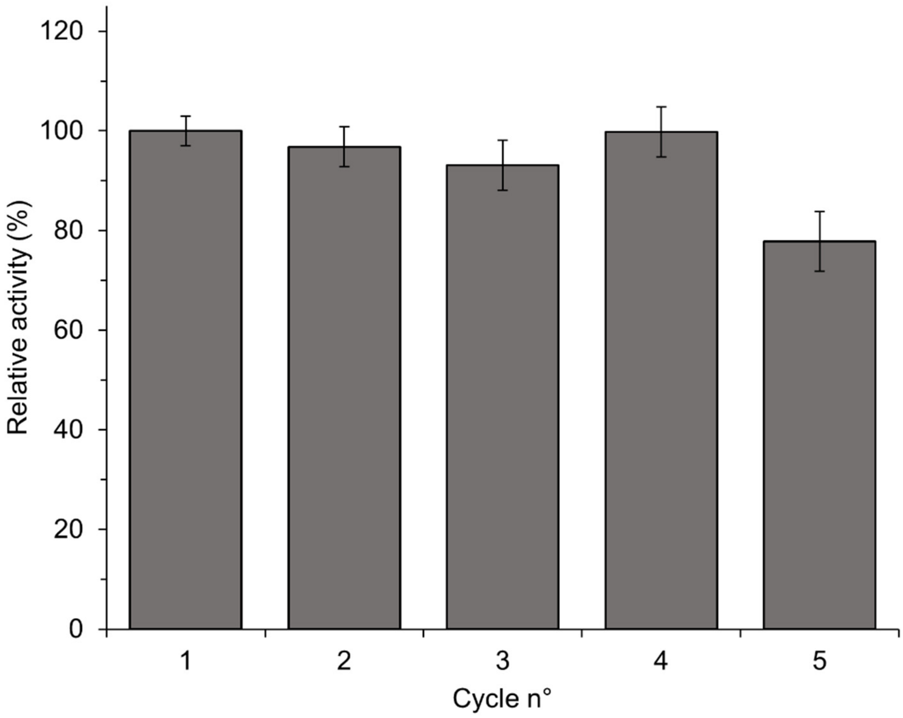

Relative activity: % conversion achieved with the recycled enzyme with respect to that obtained in the first cycle.

Figure S2: Determination of the conversion by ^{1}H NMR analysis

The conversion of **1** into the corresponding 3α-acetoacetoxy derivative **3a** has been calculated considering the integrals of a proton of **3a** with respect to that of a proton of **1**. As the integral of 1H of **3a** has been considered the integral of the multiplet at 4.79-4.69 ppm due to the resonance of the 3α-H of **3a**. The integral of 1H of **1** as been deducted as belove described from the value of the integral of the multiplet at 3.65-3.50 ppm due to the sum of the resonances of 3α-H of **1**, 7β-H of **1** and 7β-H of **3a**).

1H of **1** = (∫ m 3.65-3.50 ppm – ∫ m at 4.79-4.62 ppm)/2

Conversion % = [1H of **3a** / (1H of **3a** + 1H of **1**)] x 100

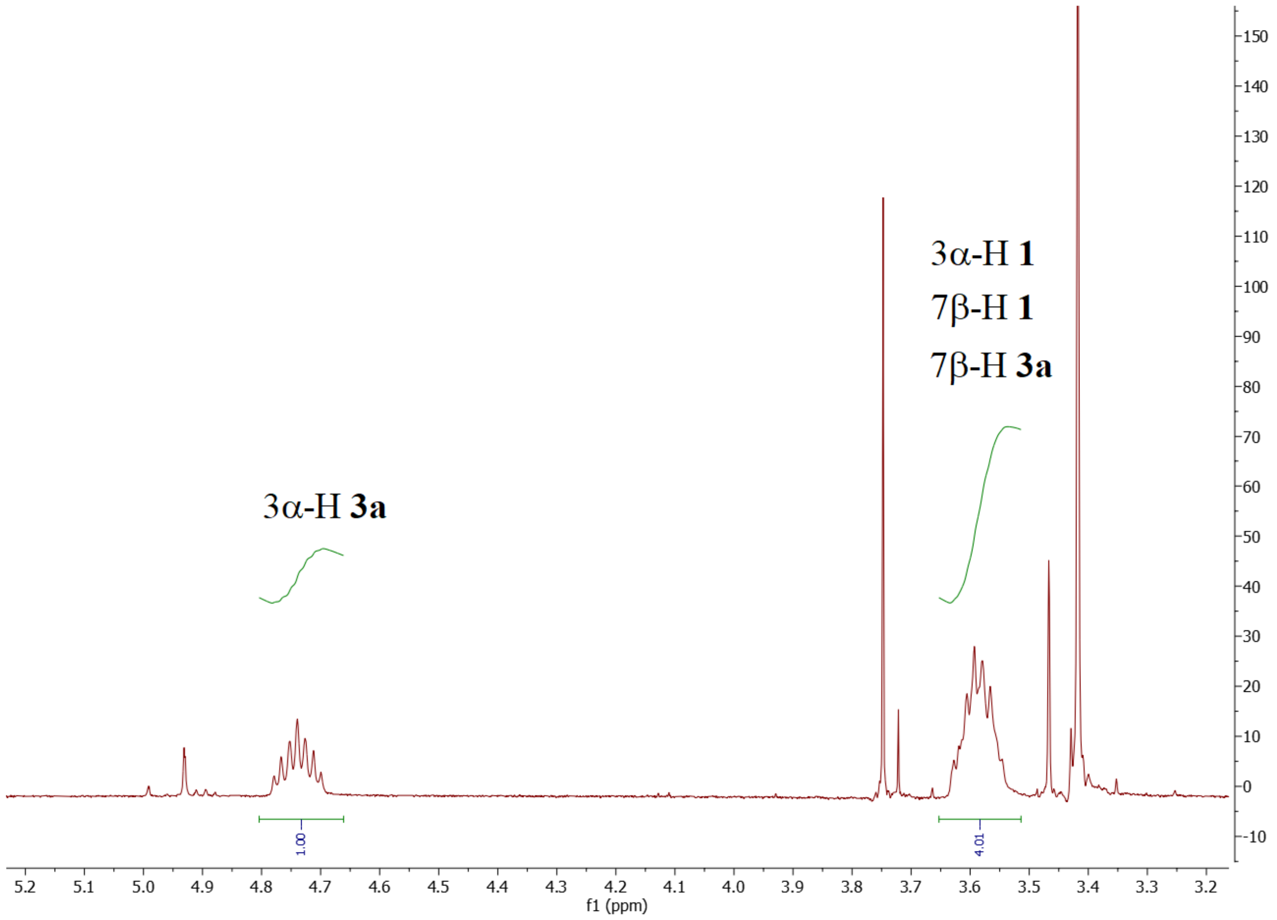

Figure S3: ^{1}H- and ^{13}C-NMR spectra of compound 3a

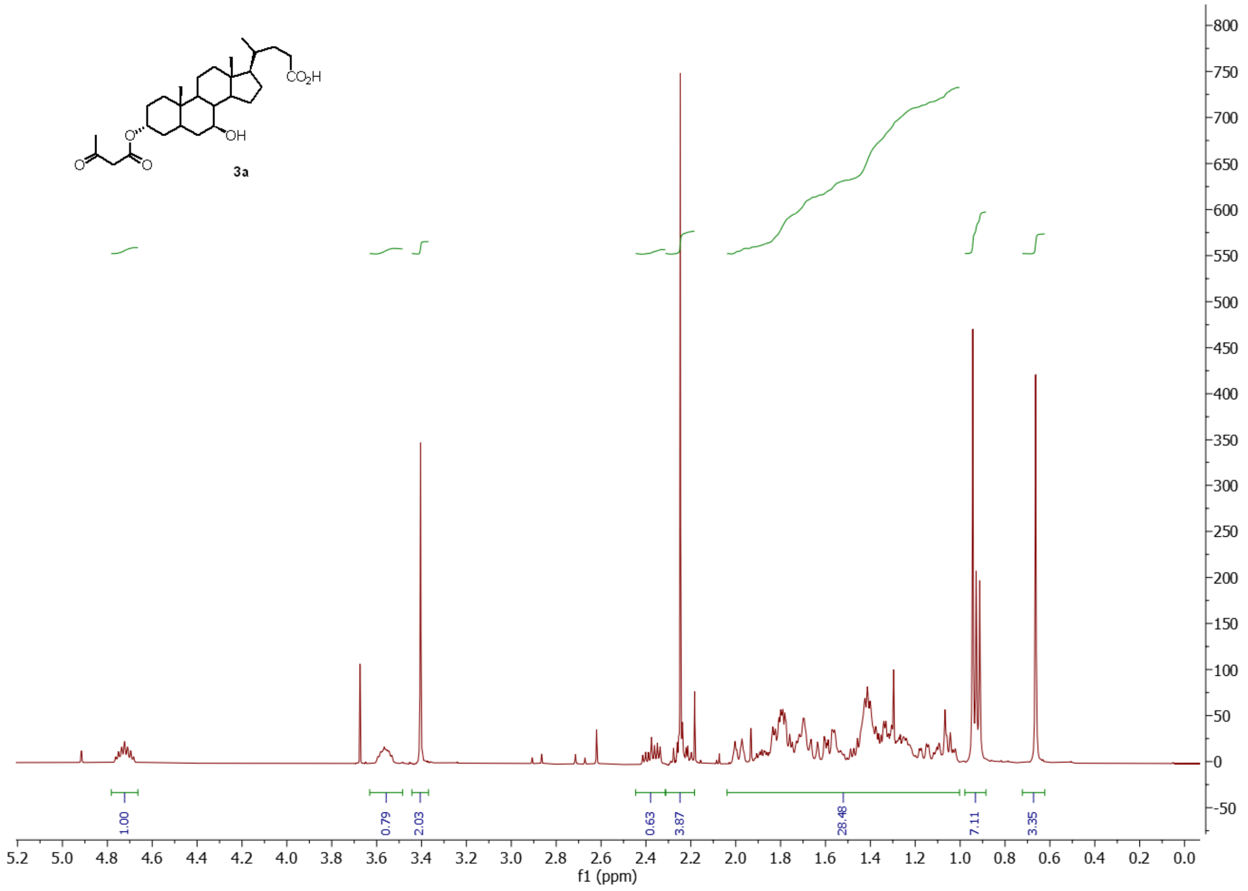

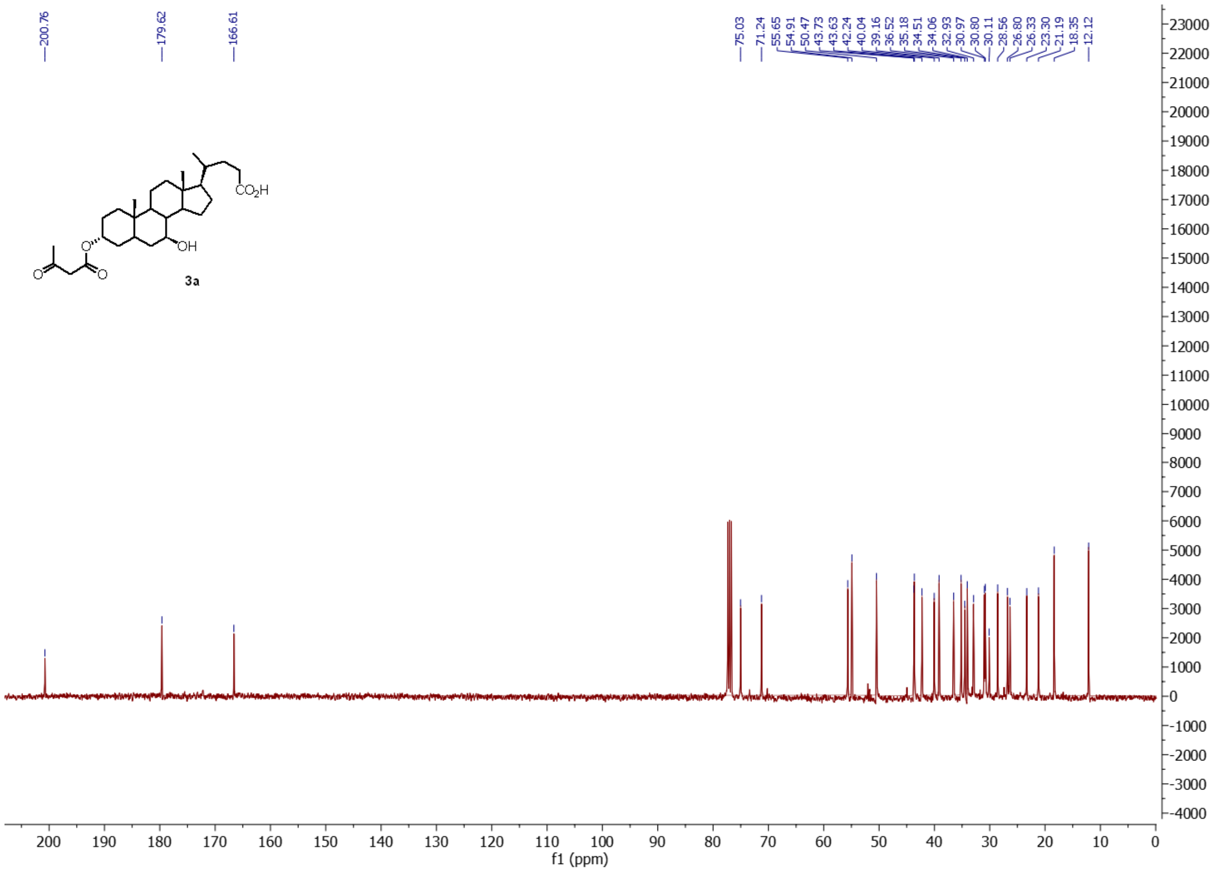

Figure S4: ^{1}H- and ^{13}C-NMR spectra of compound 3b

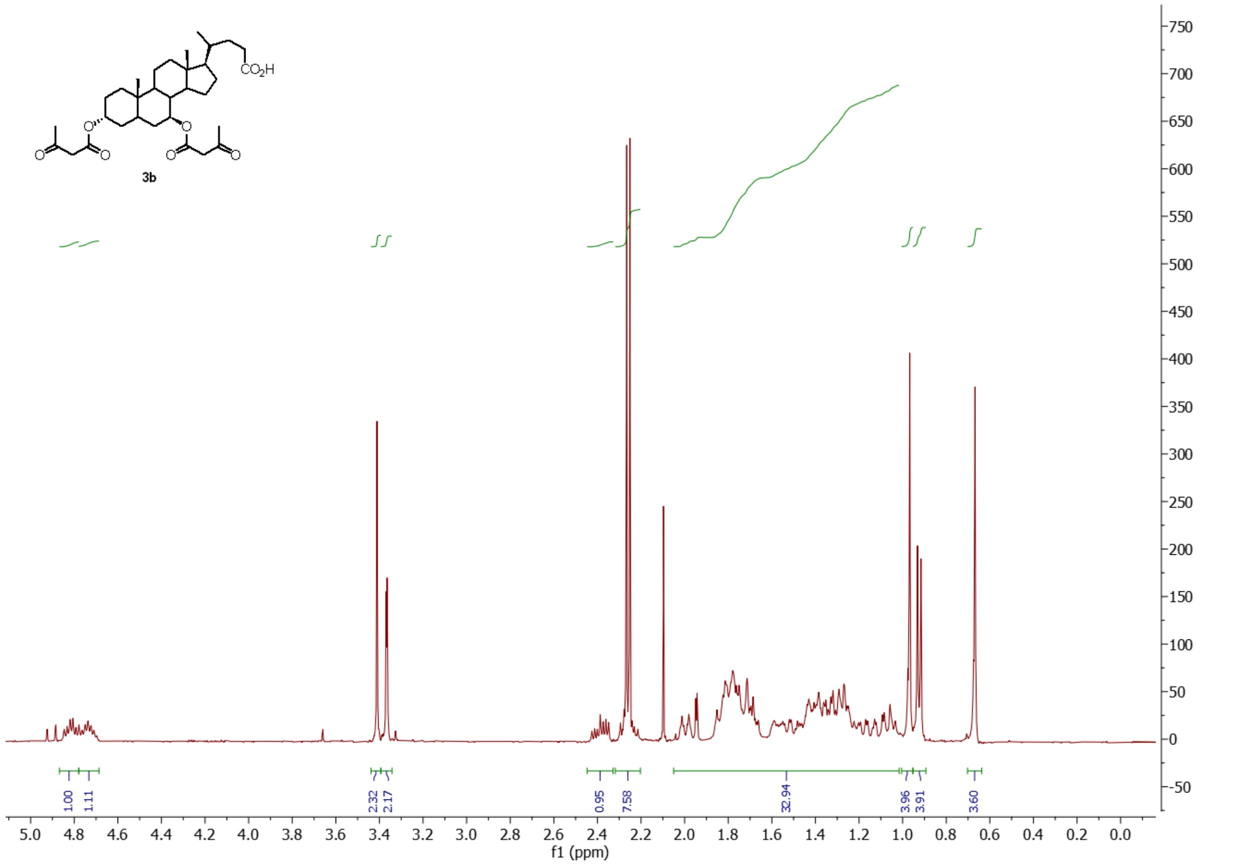

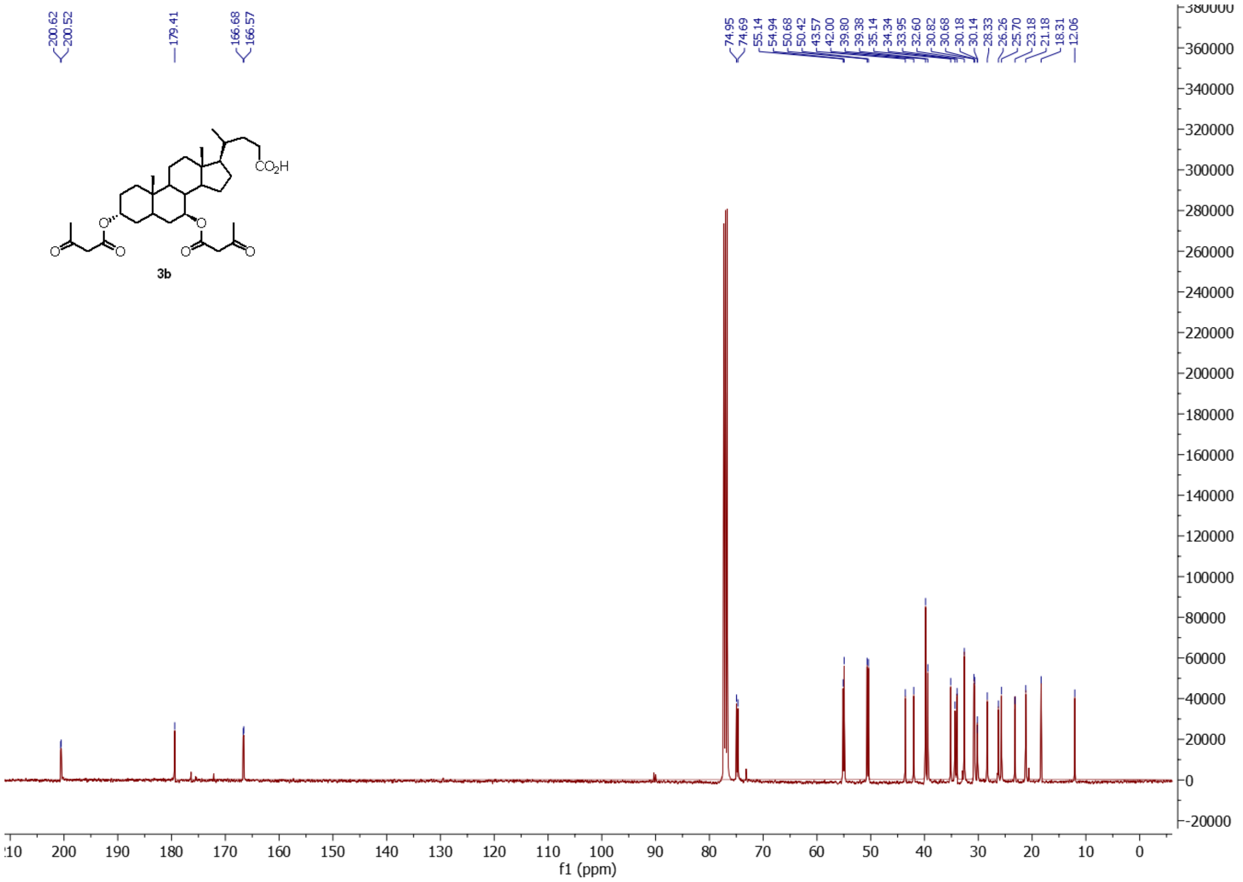

Figure S5: ^{1}H- and ^{13}C-NMR spectra of compound 3c

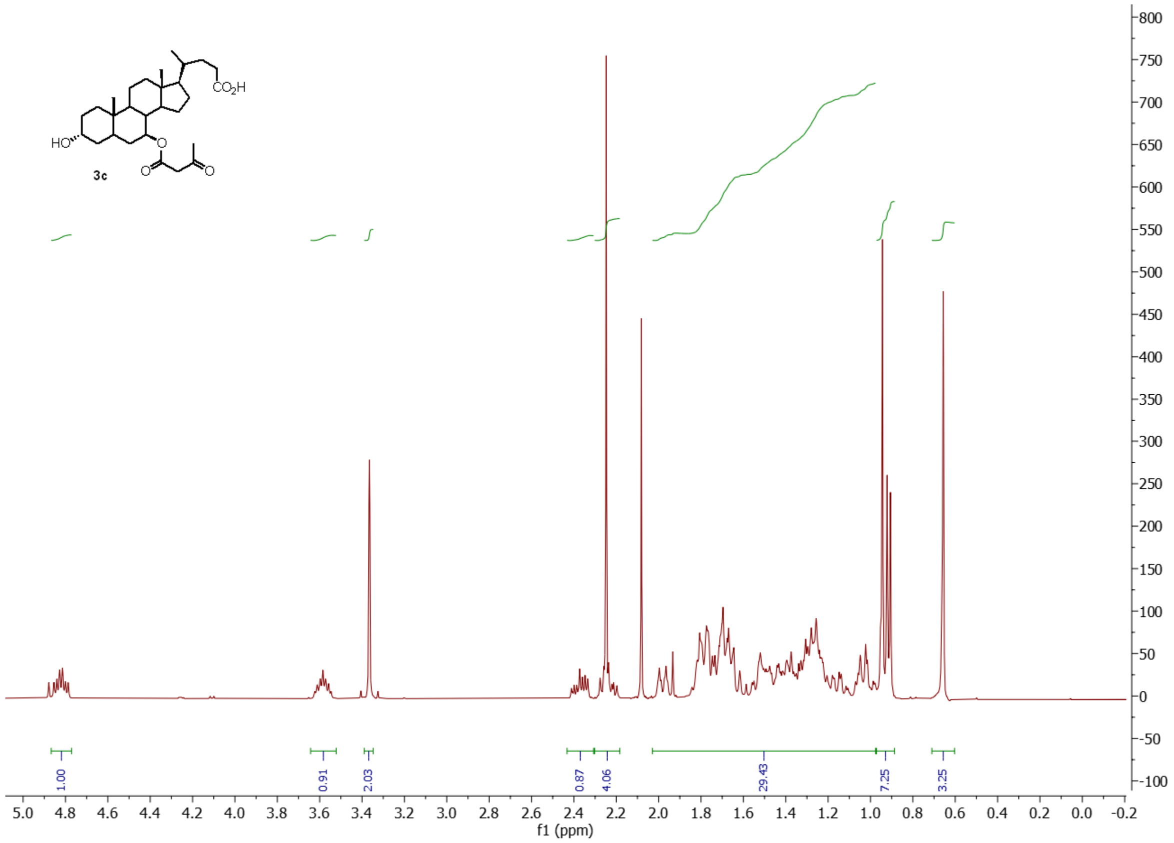

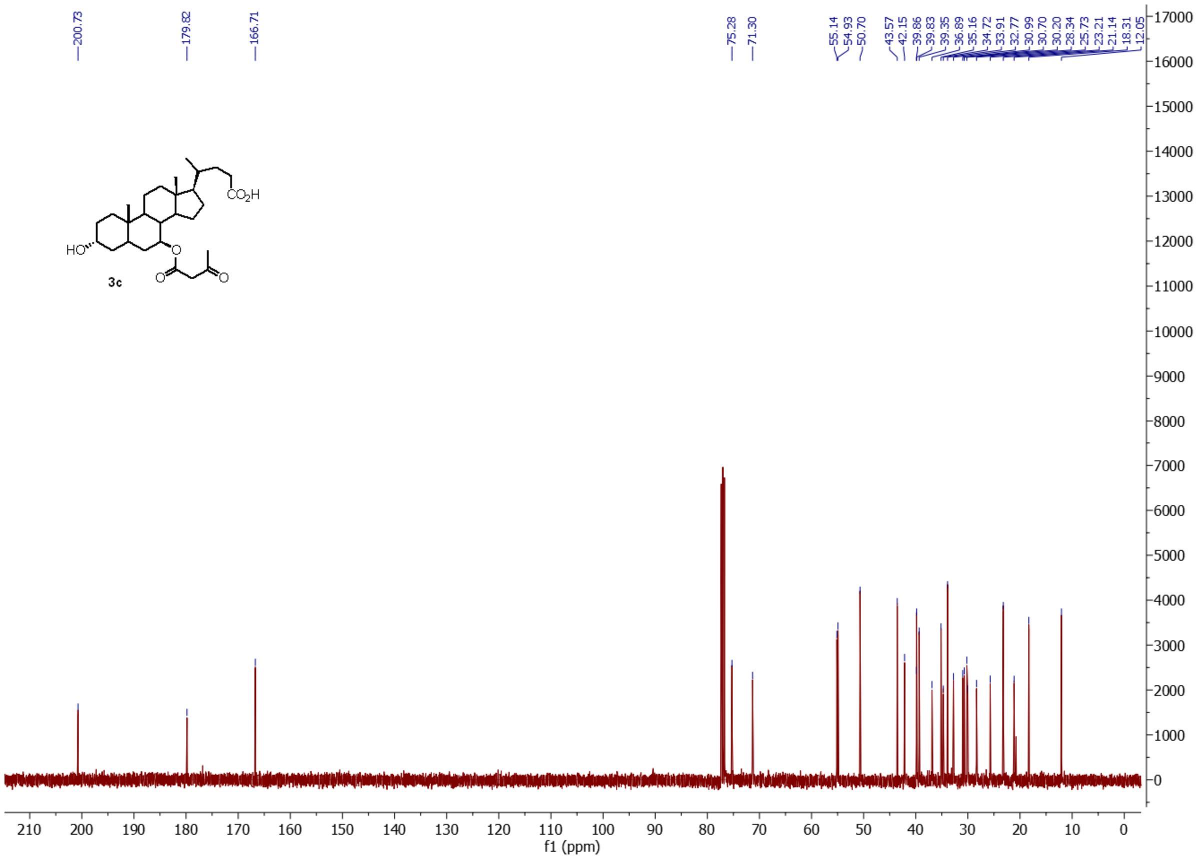

Figure S6: ^{1}H- and ^{13}C-NMR spectra of compound 4a (diastereomeric mixture)

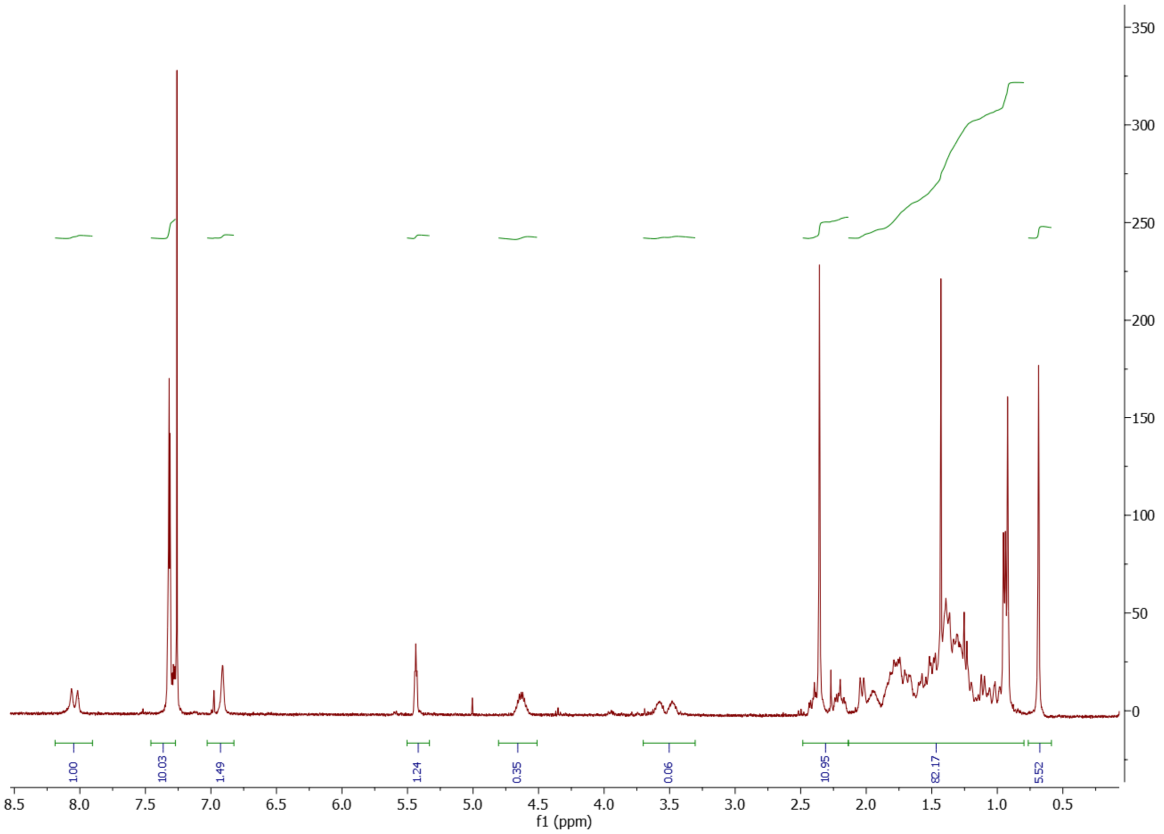

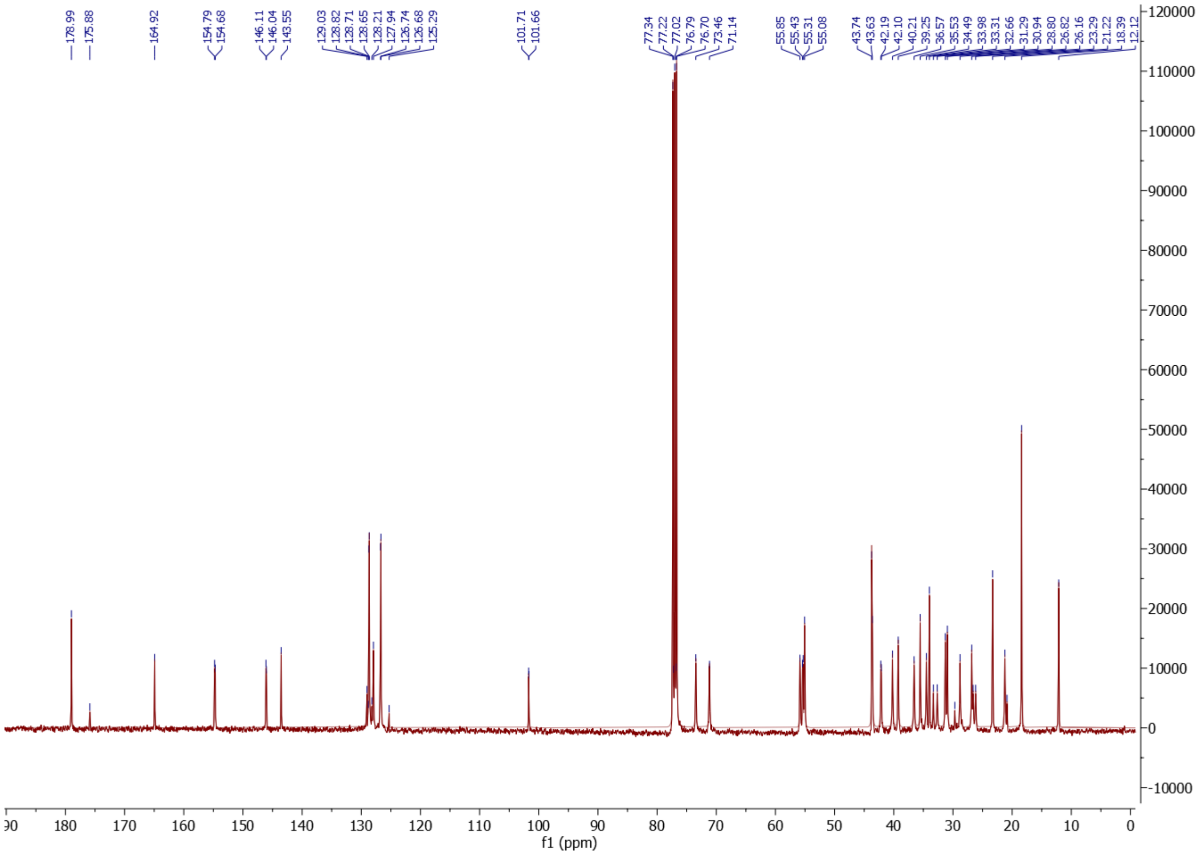

Figure S7: ^{1}H-NMR spectra of single diastereoisomers of compound 4a

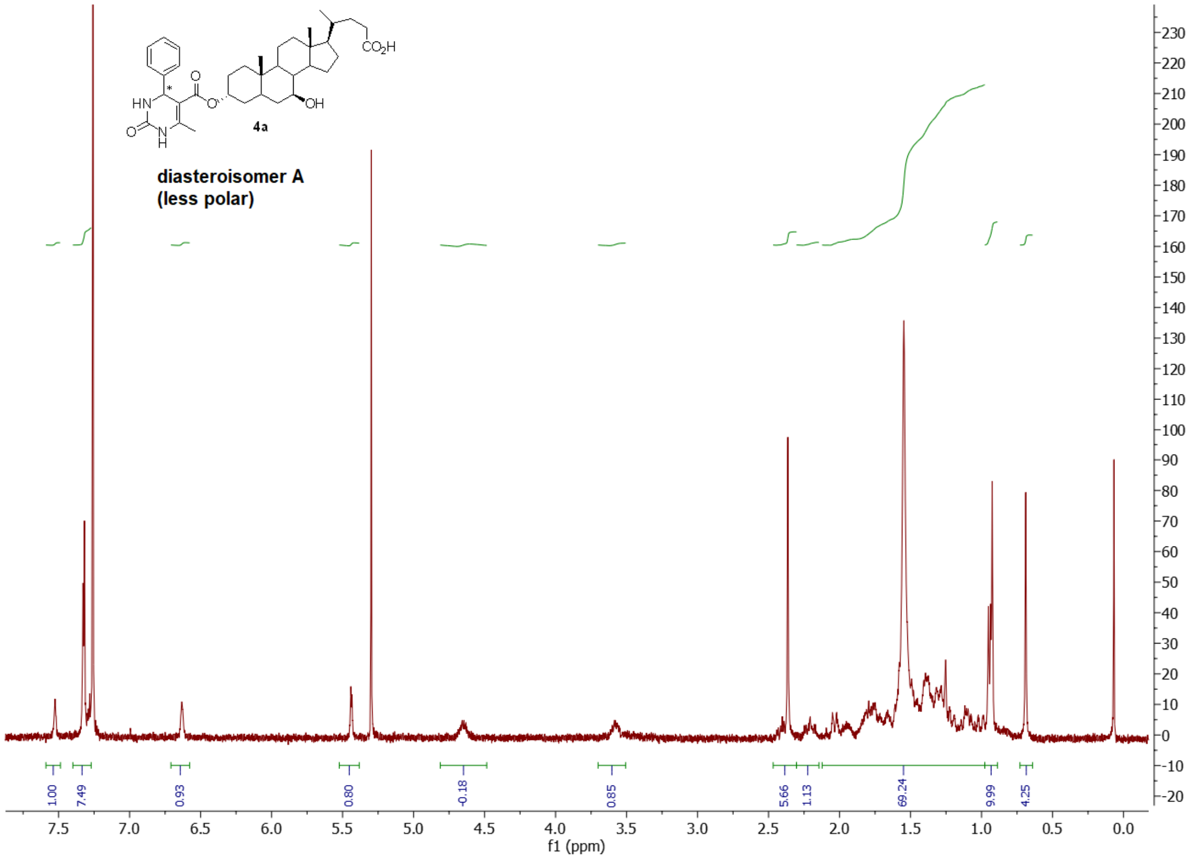

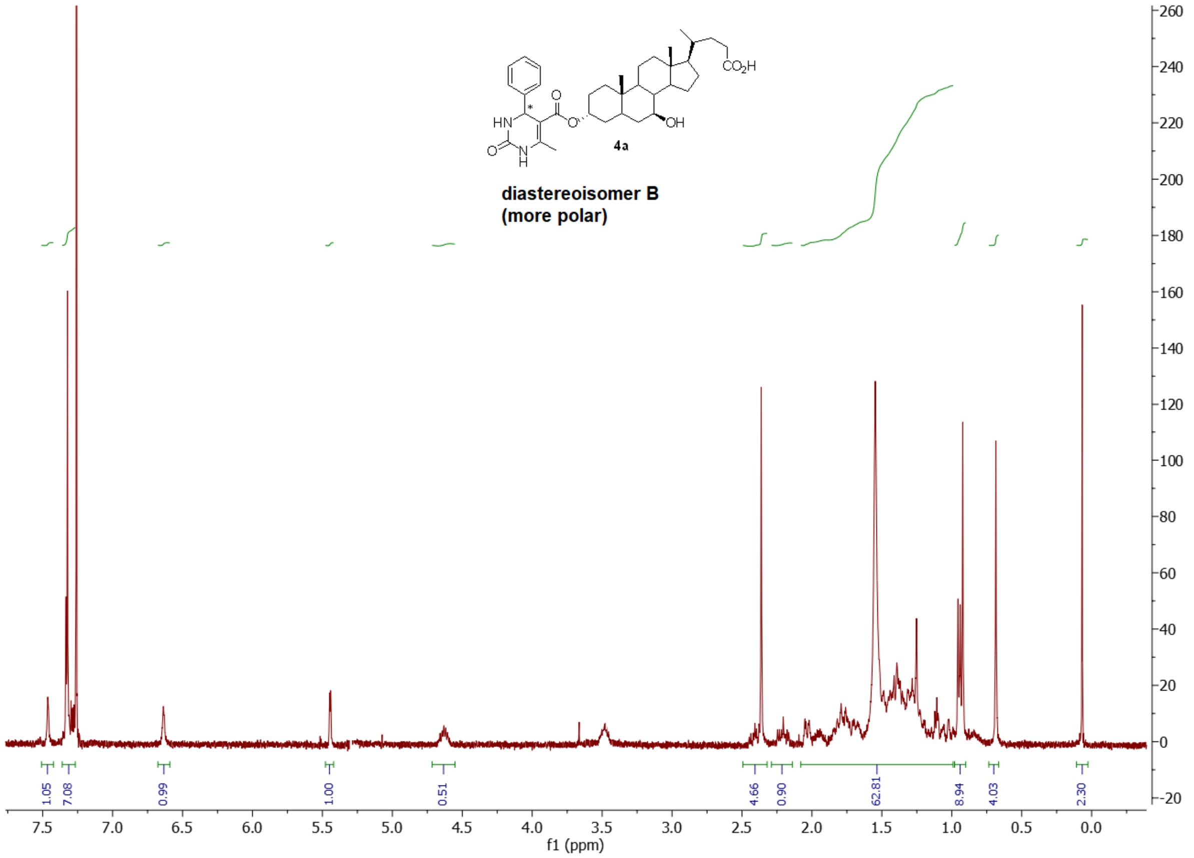

Figure S8: UPLC-MS chromatogram and HRMS of compound 3a

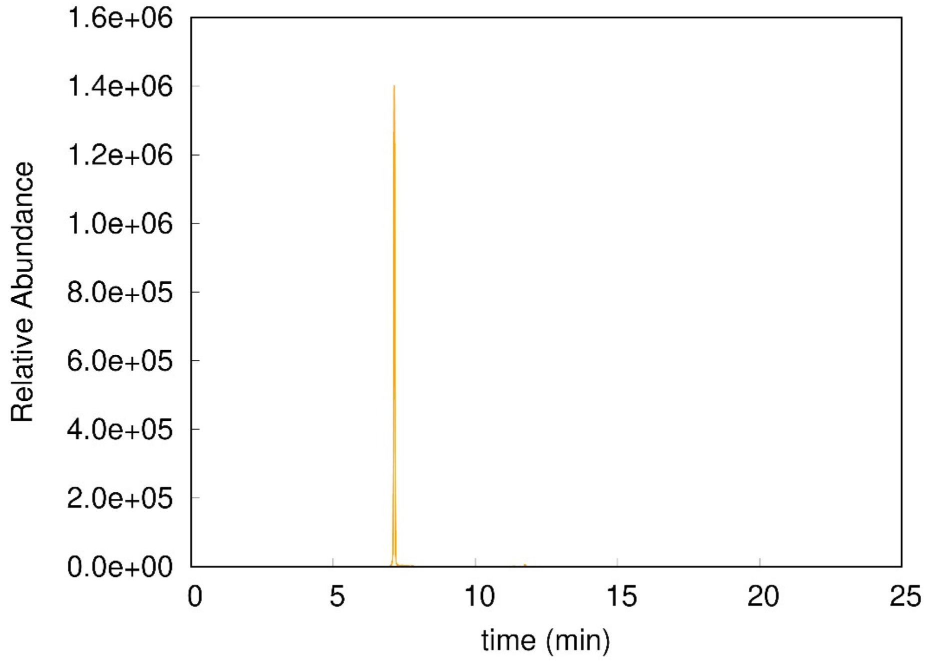

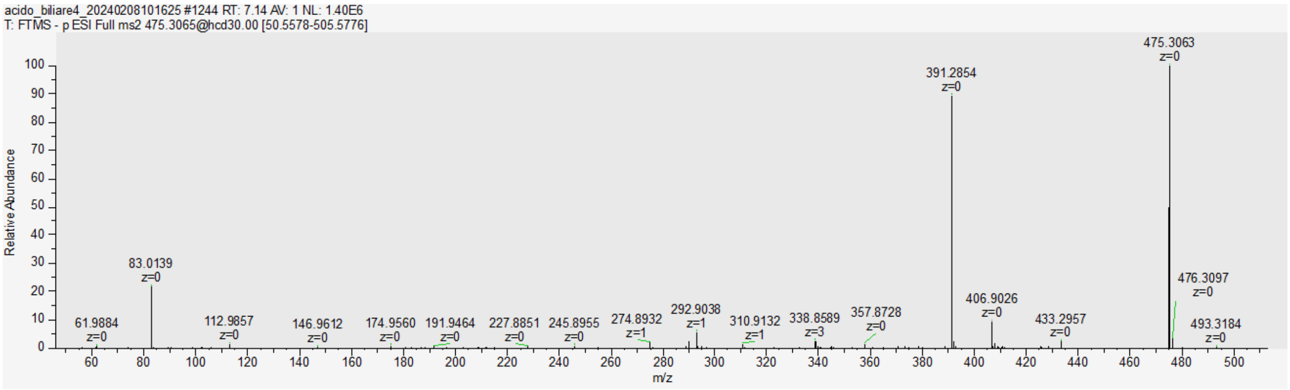

Figure S9: UPLC-MS chromatogram and HRMS of compound 3b

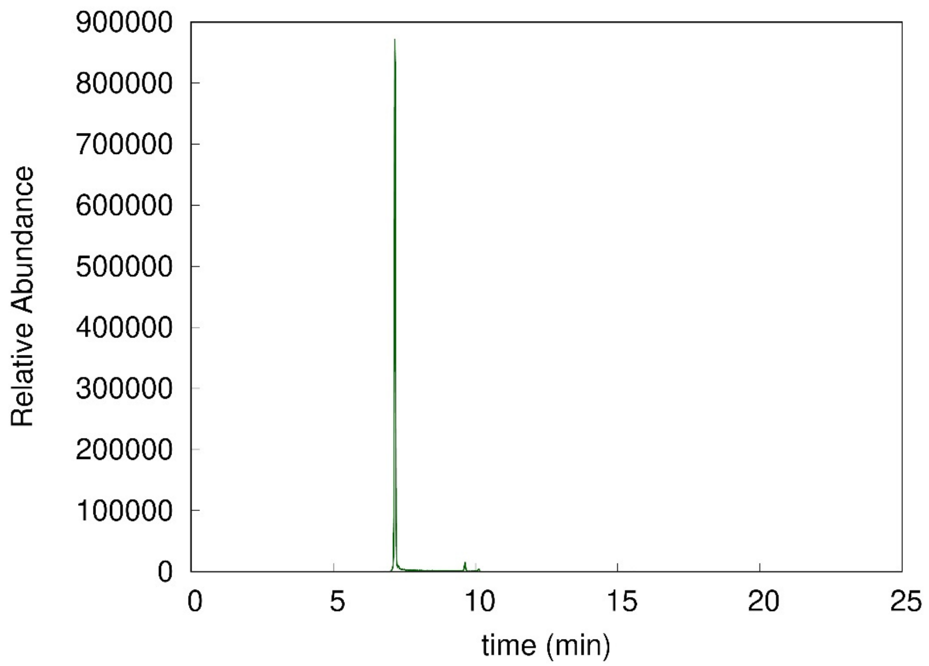

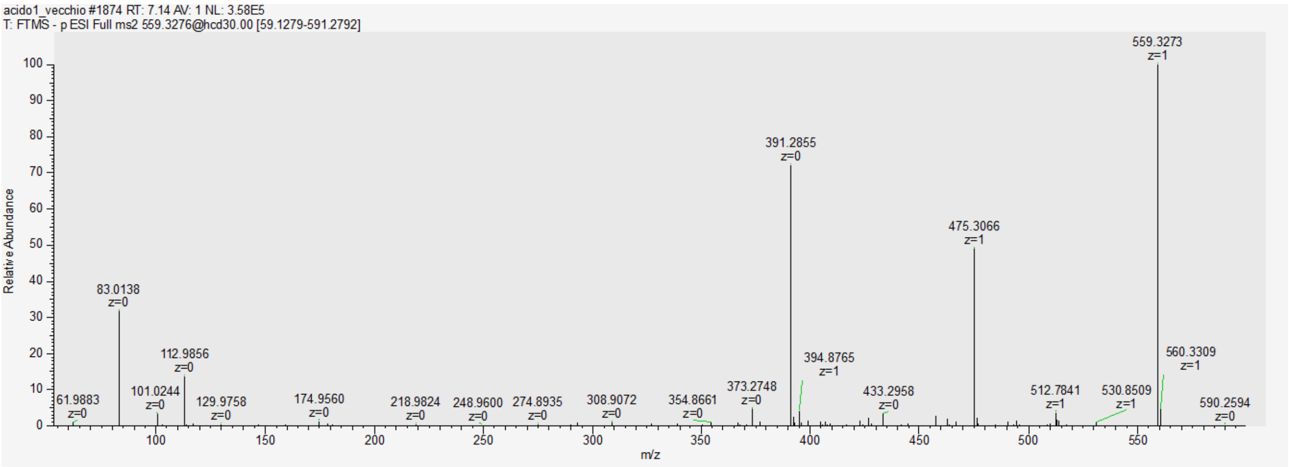

Figure S10: UPLC-MS chromatogram and HRMS of compound 3c

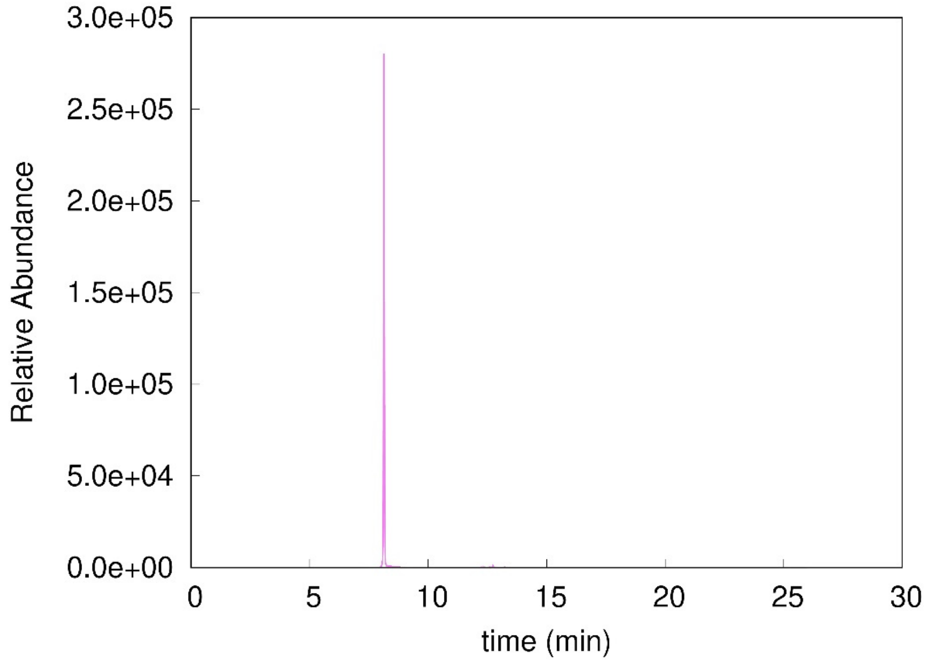

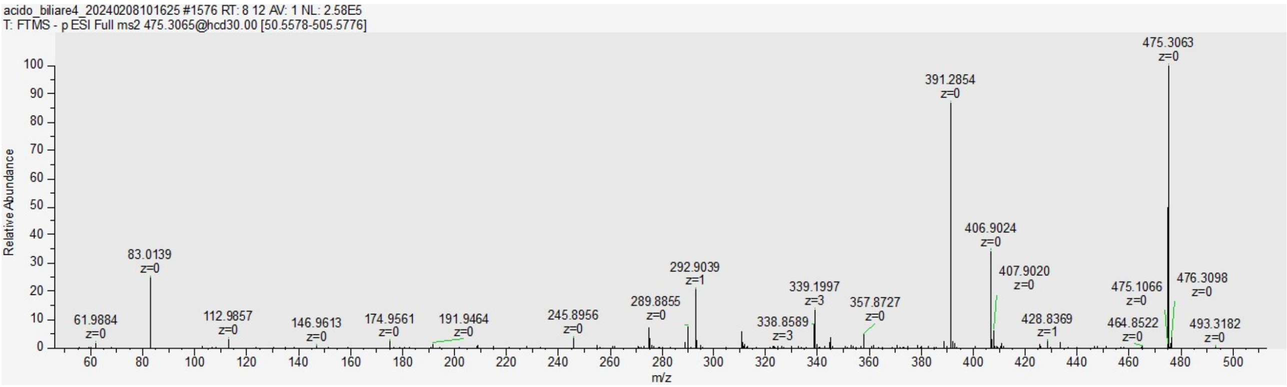

Figure S11: UPLC-MS chromatogram and HRMS of compound 3d

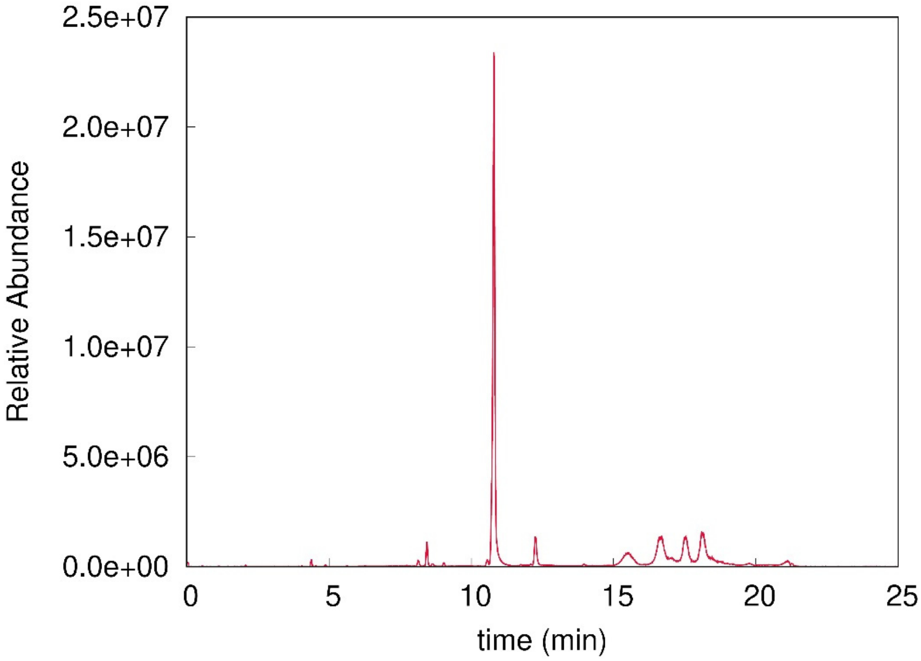

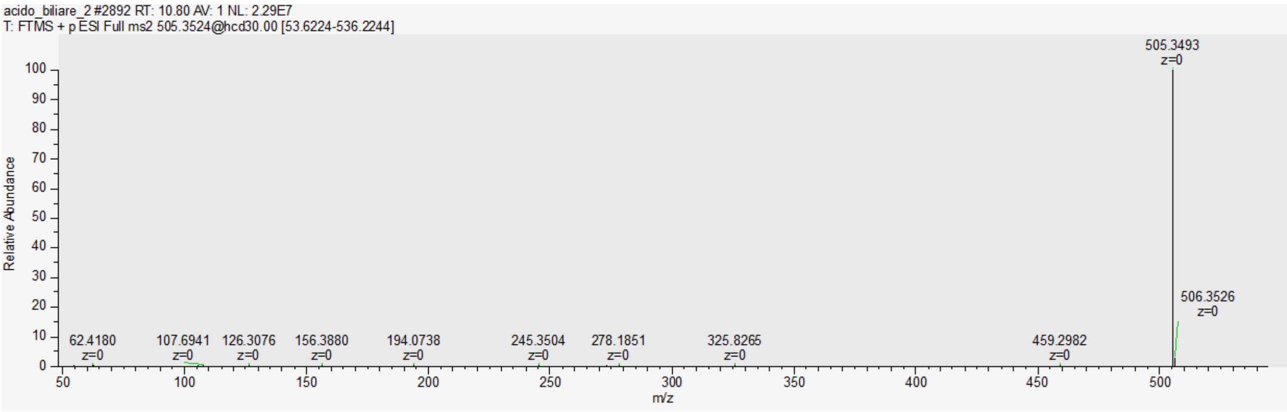

Figure S12: UPLC-MS chromatogram and HRMS of compound 4a

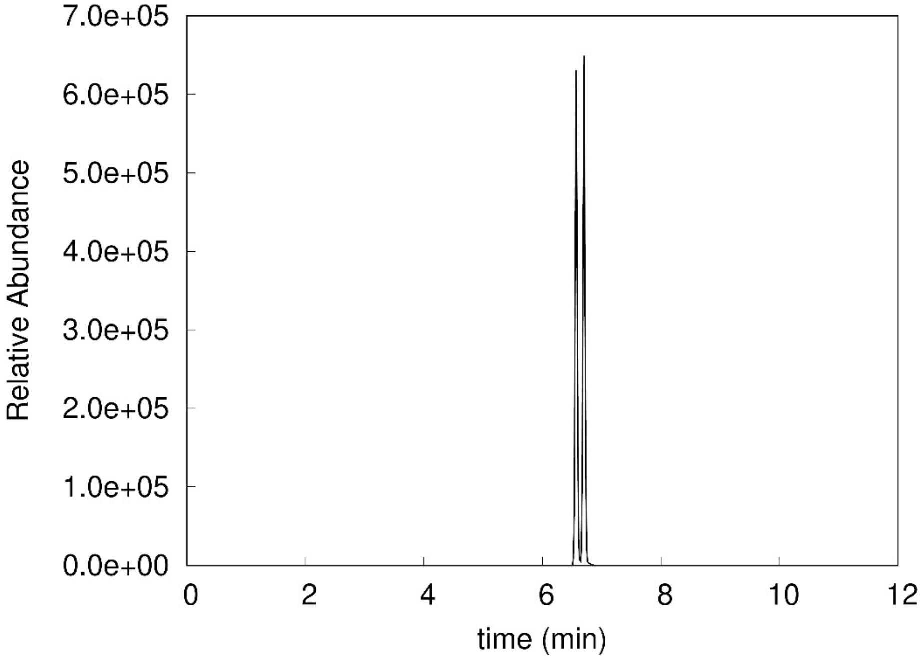

HRM spectra of the compound **4a** diastereisomer A and B (negative ion mode).

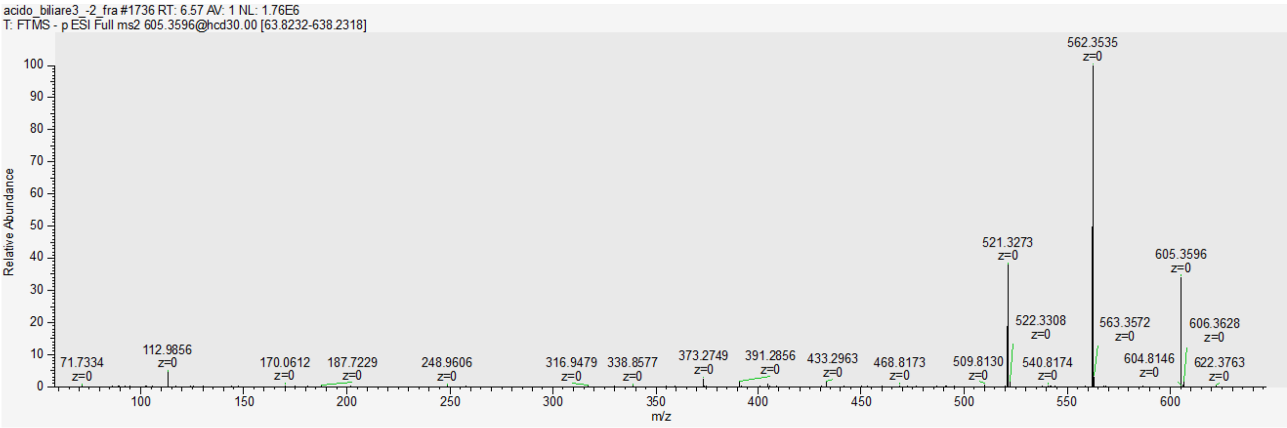

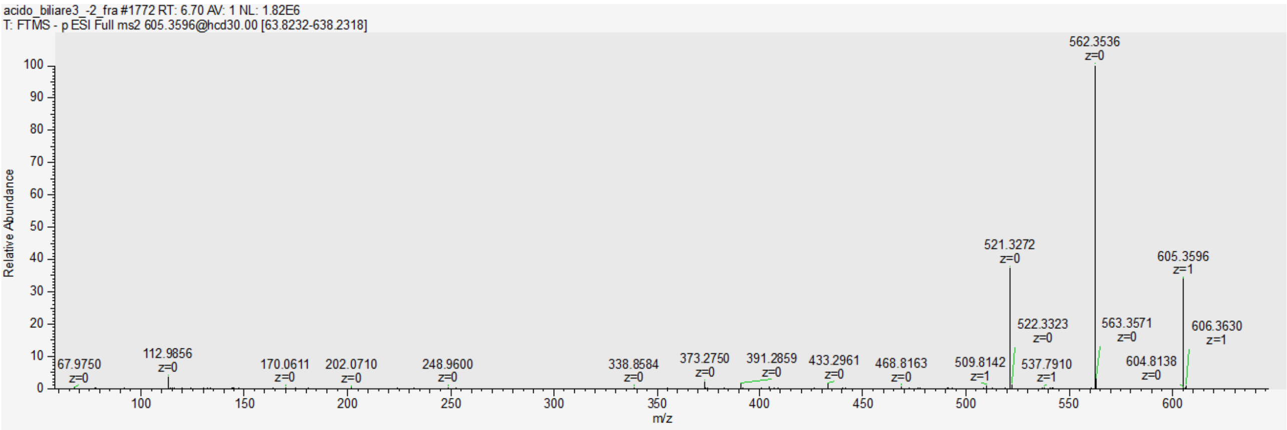

Supplement: Supplementary file 1 [file molecules-29-01305-s001.zip › molecules-2893661-supplementary.pdf]
